# Is the Lesser Khingan Suitable for the Amur Tiger Restoration? Perspectives with the Current State of the Habitat and Prey Base

**DOI:** 10.3390/ani13010155

**Published:** 2022-12-30

**Authors:** Anna Yachmennikova, Shibing Zhu, Ivan Kotlov, Robert Sandlersky, Qu Yi, Viatcheslav Rozhnov

**Affiliations:** 1 A.N. Severtsov Institute of Ecology and Evolution, Russian Academy of Sciences, Leninsky, 33, Moscow 119071, Russia; 2 Institute of Natural Resources and Ecology, Heilongjiang Academy of Sciences, Harbin 150040, China; 3 National Research University—Higher School of Economics (HSE University), 20 Myasnitskaya Ulitsa, Moscow 101000, Russia

**Keywords:** Amur tiger, carnivore conservation, China, ecological network, habitat fragmentation, HSI, least cost distance, Lesser Khingan, *Panthera tigris* altaica, SDM, tiger restoration

## Abstract

**Simple Summary:**

The Amur tiger has a status of being endangered on the world’s IUCN red list. The northwestern part of its range is situated in Russia and China, where tigers were killed by humans 50–70 years ago. To restore the tiger population within the historical range, firstly we estimated the condition of the environment there. We assessed suitability of habitats for the tiger’s prey species (wild ungulates) in the Lesser Khingan mountains (North China). For this we made modeling and calculations that were based on the information from satellite images and data we collected personally on the land surface during our expeditions. The resulting species distribution maps were used to design an ecological network. The habitat patches with the best quality (for tiger) were assigned as cores for the ecological network, which were connected by calculated green corridors. The recovery of the Amur tiger in habitats of China’s Lesser Khingan is confirmed possible. Natural green corridors for moving tigers are mainly located at the forests’ edges and characterized with high variability of the trees species. In this study, we describe three potential transboundary corridors and make recommendations to establish protected areas in the important tiger places. Moreover, it is necessary to implement habitat recovery activities for tiger key areas.

**Abstract:**

The Amur tiger (*Panthera tigris*) has a status of being endangered on the world’s IUCN red list. The northwestern part of its range is situated in Russia and China, where tigers were exterminated by humans in the 1950–1970s. To restore tiger population within a historical range, an estimation of the habitat suitability is firstly needed. The Lesser Khingan mountains (Heilongjiang) was analyzed. Habitat types were mapped by satellite images analysis and field proven. The potential habitats of the main tiger’s prey species (wild boar (*Sus scrofa*), roe deer (*Capreolus pygargus*), and red deer (*Cervus elaphus xanthopygus*) were also assessed. Maximum entropy and linear discriminant analysis methods were applied and compared for species distribution modeling (SDM). Species distribution maps were used to design an ecological network. The fragmentation of habitat patches was evaluated by spatial ecological metrics. The habitat patches with the best metrics were assigned as cores for the ecological network, which were connected by calculated corridors. The least cost distance method (based on distance to roads and settlements) was used. The recovery of the Amur tiger in habitats of China’s Lesser Khingan is shown to be possible. Types of habitats were calculated as natural corridors for moving tigers. They are mainly located at the forests’ edges and characterized with various canopy structures and high variability in the tree species composition. Three potential transboundary corridors are described: (a) foothills and low mountains of the northern Lesser Khingan; (b) connection between the southeast Lesser Khingan and the western part of the Wandashan mountain system; and (c) corridor within foothills and low mountains of the eastern part of Lesser Khingan. It is recommended to establish protected areas for the important tiger core habitats, and the main optimal ways for their migrations are described during the current investigation. Moreover, it is necessary to implement habitat recovery activities for key areas.

## 1. Introduction

Habitats of the Amur tiger (*Panthera tigris* altaica) historical range are still largely intact despite their obvious mosaic and fragmented spatial structure [1,2]. Historical range of the tiger covers mountain systems of the Sikhote-Alin, the Lesser Khingan, and the Changbaishan, as well as the Amur river valley taiga forest area (historical lands of Manchuria) [3]. Currently, these territories belong to the Far Eastern region of Russia (Amur, Jewish, and Khabarovsk regions), as well as the northeastern parts of the People’s Republic of China (Heilongjiang and Jilin provinces) [4]. The number of Amur tigers in Russia is still extremely small and unstable, which has been estimated as no more than 500 individuals in total [5,6]. In China, tigers are recorded regularly but mainly single visits are described—each registration identifies a new individual, that was not described earlier (15–30 different animals; [7]). These data are mainly related to the cross-border region of the Russian Far East and the Jilin Province (the People Republic of China—PRC) and their border with Korea [8,9].

Within the historical range, the number of tigers sharply decreased in the 1950–1970s due to the direct killing and poaching of these animals by humans [4]. An intensive forestry and agriculture activity in that region (both in the USSR and China) was carried out during that period. Currently, the tiger is listed in the Red Book of the Russian Federation [10], and in the China Red Data Book of Endangered Animals [11]. The International Union for Conservation of Nature (IUCN) red list puts the Amur tiger status as “endangered” [1,2].

Several special programs designed to preserve the tiger have been implemented within its Russian range. Nevertheless, the most effective work should include cross-border activities and should be implemented not only in Russia but also in China, within the natural boundaries of the tiger’s distribution, regardless of political borders. Such practices are successfully implemented in Europe, South America, and Southwest Asia [12,13,14,15]. The Lesser Khingan mountain area has a high potential probability of tigers inhabiting it [16]. The successful implementation of the A.N. Severtsov Institute of Ecology and Evolution, Russian Academy of Sciences (IEE RAS) project of the tiger’s restoration in the northwestern part of the range [3,17] also gives hope for tiger recovery in China. It was confirmed that two of the six tigers released in Russia had visited forests of the Chinese Lesser Khingan (Heilongjiang Province). The cats crossed the Amur River (separately) and explored territory in China during the two month period and then returned to Russia [3,17]. The Russian experience of animal reintroduction is next: six individuals were released in 2013 and 2014 and then they adapted successfully within the territory where wild tigers have not been registered for more than 40 years. Thus, these released six individuals successfully survived; five of them established individual home ranges and started to breed in the wild. This tiger populational grouping developed and currently has no less than 15 tigers [3]. Thus, the recovery of the tiger in the north of China could be both implemented by using the Russian experience of animal reintroduction [3,17] or as a result of natural migration, movement, and spread of young animals (the offspring of restored tigers in Russia) to the South.

Despite the way of the population restoring, it is necessary to have the most complete picture of the currently available habitat structure for potential area which is planned for tiger restoration. The current Russian–Chinese cooperation program is aimed at full recovery of tigers within the Lesser Khingan [18]. Habitat assessment is important to create official ground for the organization of new protected areas and to identify areas where human influence should be minimized (Figure A5). The areas which are necessary to be restored and recovered (from positions of habitat fragmentation) currently need the strengthening of the protection measures and changes in environmental management regimes. Such kind of work anticipates the start of any big complex project aimed directly at working with rare mammals with the further goal of restoring their populations. Moreover, it allows to retrospectively understand the spatial dynamics of natural systems within the species habitat (Chile biodiversity—[19]; Nepal tigers—[20]), it can be further used for an ecosystem process of forecasting and planning. In particular, it is necessary grounds for developing action plans and updating environmental programs.

Such evaluations are relevant everywhere due to the intensive reduction and destruction of suitable habitat areas for a number of species in all countries of the world; for bustards in Spain [21]; carnivorous mammals in North America, including lynxes, bears, and wolverines [22]; lemurs in Madagascar [23]; Persian leopards in the Russian Caucasus [24,25,26,27,28]; cougars in middle and western America [29]; lynxes in Switzerland [30]; small cats in Eurasia [31] and the Southeast Asia [32]; tigers in India and Nepal [20,33,34,35,36]; and tigers in Sumatra [37]. For the eastern and northeastern parts of China, as well as the territories of the Russian Far East, similar studies on the Amur tiger have also been repeatedly carried out by various researchers [5,38]. There is a knowledge gap in the evaluation of suitable habitats for Lesser Khingan. Another important feature of protected species ecology relates to their migration, including diurnal, seasonal, and year-round movements within and between habitats. Many investigations devoted to the tiger migration are focused on features of space use and movements of these animals [39,40,41,42,43,44]. A network of routes is the main characteristic that could describe the structure of individual animal sites (home ranges) [45]. Spatial and temporal (network) analysis is an important part of the large-scale monitoring [24], as it considers the spatial distribution of the tiger prey base [46]. Such analysis can take some years due the fact that individual tigers can use areas up to 80–100 thousand hectares during the process of home site establishment [24], especially during the period of younglings’ resettlement. The natural space that animals use is not uniform in its good or bad qualities. Areas that tigers pass through when moving may even include human-used habitats that are insufficient and unsuitable for tiger breeding, hunting or resting [47], but are comfortable enough for migrating activity. These features must be considered when assessing the tiger’s potential habitats. First, the key zone areas should be detected (core areas). They become a base for the existence of stable animal groupings. This is why the pathways and transit zones between key zones (the ecological corridors) should be identified.

Properly designed transit zones provide animals with the possibility to migrate over long distances of tens or hundreds of kilometers, even in regions with high human population density and a dense infrastructure network. Systematically supported ecological corridors allow for a meta-population establishment. This approach is especially important for maintaining genetic diversity [37].

The purpose of our work was to assess the potential suitability of the Lesser Khingan for the further restoration of the tiger population and to design a potential ecological network for the Amur tiger and its prey ungulates, as a system comprising ecological cores and corridors. Our objectives were to (1) identify potentially suitable habitats for the tiger in the Lesser Khingan mountain system area (-habitat types description;—mapping the quality of prey species habitats;—ranking of prey habitat types for tiger suitability); (2) analyze fragmentation and to identify potentially suitable ecological cores and corridors for the Amur tiger and its prey base by means of species distribution modeling (SDM) and GIS analysis; and (3) analyze the current system of protected areas and to compare it with the resulting network of the key territories for the tiger.

## 2. Materials and Methods

The concept of spatial ecological niche was used as the methodological ground for assessing the suitability of habitats. This concept supposes the ecological niche of a species exists as a spatial domain within the multidimensional space of environmental factors. Species survival and reproduction are possible here [48,49,50]. Spatial ecological niche can be evaluated through the SDM and habitat suitability index (HSI) evaluation [51]. The complexity of spatial ecological niche models depends on the number of environmental properties that can be quantified. In recent decades, the SDM and HSI have been largely formalized (BioGeomancer (BioGeomancer Working Group), DesktopGarp, WhyWhere (Central Queensland University, Australia), Biomapper (University of Lausanne, Switzerland), etc.). Publicly available tools include software for modeling based on environmental data sets (remote sensing data, soil data, geobotanical data, other ground cover data, and climate databases). Large-scale studies (population scale) use remote sensing data with a resolution of n*10^−1^ n*10^1^ m. Remote sensing data characterize properties and conditions of habitats (composition and state of vegetation, biological productivity, etc.) indirectly through spectral reflectance. Digital elevation models (DEM) characterize the redistribution of heat and precipitation by the landscape. Wide range of statistical methods are used in SDM as basic (regression, discriminant analysis) and supplementary (distribution evaluation, outliers removal etc.) techniques [52,53].

Current study further develops our previous case study that was implemented for the Taipingou National Park (TNP), China [18], which is situated on the northeast of the Lesser Khingan.

### 2.1. Study Area

**Figure 1:**
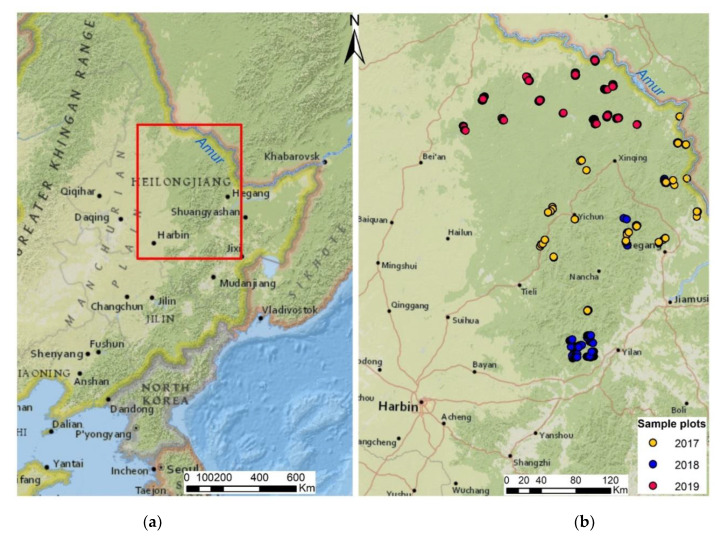
(**a**) Map of the study area. The Lesser Khingan mountain system is located in the north central part of Heilongjiang Province, China and borders Russia in the northeast, rectangle – study area; (**b**) map indicating field collection sites.

Geographical characteristic. The Lesser Khingan mountains are one of three transboundary mountain systems that cross the Amur valley in its middle flow (the meridional direction of the Bureya Ridge) [54]. It is situated between 45° N 125° E and 50° N 131° E and is located in the north central part of Heilongjiang Province, China (Figure 1a), bordering Russia in the northeast. [55]. The highest peaks are situated in the southeast and lowest peaks—in the northwest. The average altitude is 500–1000 m and the highest altitude is 1429 m above sea level. The Lesser Khingan is characterized by smooth, wavy relief; it is a system of wooded mountains and plateaus. The rivers are characterized by gentle, slightly incised valleys or marshy valleys with steep slopes in their middle part. The climate is humid and belongs to the continental monsoon climate area of the North Temperate Zone, with an annual average temperature of −1 + 1 °C, four distinct seasons and a frost-free period of 100–120 days [55].

Floristic description. The flora and the landscape here are similar to the adjacent parts of the East Manchurian Mountains. The study area belongs to the East Asian floristic region of the Manchurian floristic province [56] with a predominance of forest vegetation. The Manchurian flora is the richest and most diverse (in comparing with Daurian flora); it covers the East Manchurian mountains, the Ussuri-river basin, and lasts along the middle flow of the Amur River within the Lesser Khingan mountains; it is characterized with large number of thermophilic and relict forest plant species (are common in the subtropics and, in part, even the tropics of East Asia). Thus, the flora of the Lesser Khingan mountains is complex and diverse; it includes north temperate flora mixed with subtropical, tropical, and cold temperate flora.

Faunistic description. Dominant species of ungulates are the wild boar (*Sus scrofa*) and the roe deer (*Capreolus pygargus*). The red deer (*Cervus elaphus xanthopygus)* should naturally be widely spread here but has rarely been registered in recent times. In 1975, the population of red deer in the Yichun forest area of Lesser Khingan mountains was approximately 7500 individuals; in 1990—5147 individuals [57]; and in 2000—3363 individuals [58]. Recently, there has been a lack of data, but the numbers show a downward trend. The encounter rate of red deer footprints in our survey is also very low. The dominant and common species of carnivores are the weasel (*Mustela sibirica*) and the yellow-throated marten (*Martes flavigula*). The lynx (*Lynx lynx*) and the leopard cat (*Prionailurus bengalensis*) are rarely registered. Some species, such as the Amur tiger and sika deer (*Cervus Nippon*), have disappeared here due to human disturbance and habitat disturbance.

People population density. Yichun city area—is the main and biggest subregional part of the Lesser Khingan mountain area. According to official data, the decrease in the Yichun city population was 269,245 (23.45%) from 2010 to 2020 [59]. The current population of Yichun is still huge (878 881, which is approximately 80% of all the population of the Lesser Khingan). At the present time, wild-habitat disturbance mainly come from understory planting (cultivating medicinal herbs, such as Ginseng in the shade of forest-trees, without cutting down trees by locals, etc.), cattle grazing, and wild berry/flower/fruitage picking and poaching. In December 2019, Yichun city region had a total land area of 3.28 million hectares. It includes 259,000 hectares of cultivated land—7.9% of the total land area is busy with cities; the garden area is 1880.42 hectares, 0.06%; 2.8507 million hectares of forest land, 86.91%; and the grassland is 26,300 hectares, 0.8% [60].

### 2.2. Study Design

The study includes the following stages. The sample plot data (identification of forest types and ungulate distribution) were collected during the field periods. The field data were classified into different habitat types. Habitat types were further used as learning samples for habitat type mapping. The modeling of spatial distributions of three species of ungulates (red deer, roe deer, and wild boar) was carried out. They were then integrated into one prey-based HSI model of tiger. Two modeling approaches were applied and compared: maximum entropy (MaxEnt 3.4.4 (American Museum of Natural History, New York, NY, USA) and linear discriminant analysis (LDA). Finally, the design of the ecological network, including the core areas and corridors, was carried out. The proposed ecological network was compared with the current system of protected areas of Lesser Khingan (see below).

### 2.3. Field Data Collection and Pre-Processing

To obtain the vegetation types and animal distribution data, we investigated the Lesser Khingan mountains during four sessions implemented in three winter seasons (Figure 1b): 23 February–8 March 2017, 13–30 January 2018, 8–15 March 2018, and 20 February 2019–15 March 2019. Prior to the field work, we stratified the study area into different landscape conditions (strata) by using remote sensing data: multiseasonal satellite images, DEM, as well as geobotanical maps of the study area. Within each strata, we planned closed sampling routes (rectangle) 3–4 km long (optimized for one field-working day). The distance between sample routes is no less than 2 km. The route locations were chosen by time and infrastructure logistic limitations.

The data on animal footprints and vegetation conditions were recorded along the route and fixed as points with field descriptions with GPS coordinates. The data collection design on sites of field descriptions is provided in Table 1. The amount of collected points is provided in Table 2.

The vegetation type data included a description of the tree species, tree species composition (proportions of certain tree species in forest canopy), and canopy density (sparse forest or dense forest). Fifteen tree species were registered in the upper storey of forests and used for assessment of tree species composition. Among them, the broadleaf and small leaf species included oak (*Quercus mongolica*), linden (*Tilia mandshurica*), maple (*Acer* sp.), ash *(Fraxinus mandshurica)*, walnut (*Juglans mandshurica*), amur chokecherry (*Prunus maackii/Padus maackii*), amur cork (*Phellodendron amurense*), elm (*Ulmus* sp.), birch (*Betula ermanii*), alnus (*Alnus sp*), aspen (*Populus sp*); and coniferous species: pine (*Pínus koraiensis*, *pinus sibirica*), larch (*Larix amurensis*), fir (*Abies nephrolepis*), and spruce (*Picea koraiensis*). The species in the understorey and shrub layer were also recorded. The sparse forests’ type and non-forest habitats were recorded additionally as agricultural lands, wetlands, settlements, and water bodies.

As the investigator moved along the route, changes in habitat type were recorded and the location was registered. In addition, the ungulate footprints and/or faeces were identified on the snow surface during the route and recorded including GPS coordinates.

### 2.4. Remote Sensing Data Preparation

A set of environmental variables derived from remote sensing data were prepared for vegetation mapping and species distribution modeling (Table A1) The Landsat-8 Level 2 Surface Reflectance Product was used (downloaded from https://espa.cr.usgs.gov/, accessed on 1 September 2020). The study area is covered by multiple Landsat scenes related to 3 satellite paths and 2–4 rows. Due to high cloud coverage, especially in the central part and near the Amur River, the scenes belonged to different dates. We merged scenes into two Landsat mosaics: September (8 scenes—30 September 2018, 25 September 2017, 29 September 2015) and June (12 scenes—4 June 2019, 1 June 2018, 4 June 2016, 23 June 2017, 14 June 2017, 22 June 2014, 13 June 2014). Mosaic preparation included manual removal of clouds and shadows and final color balancing. The total area of Landsat mosaic covered an area of 22 million hectares. Bands 1–7 were used in modeling as well as spectral indices NDVI (normalized difference vegetation index), EVI (enhanced vegetation index), MSAVI (modified soil adjusted index), SAVI (soil adjusted index), NDMI (normalized difference moisture index), NBR, and NBR2 (normalized burn ratio) (Table A1) [56,57].

Here we are focused on the potential habitats of the tiger’s prey species and Landsat-8 images are suitable for our tasks. Landsat-8 vegetation indices are effective measures of surface vegetation [61]; they can reflect the composition and growth status of vegetation under certain conditions, which have impact on ungulates.

To remove autocorrelation, we performed principal component analysis (PCA) and used the first 6 PCA layers (99.94% accumulative of Eigenvalues). We also used digital elevation model (DEM) SRTM DEM 1 arcsecond (modified to 30 m resolution) and additionally calculated 3 topographic coefficients: slope, shaded relief (insolation from south), and root mean square (RMS) error [62,63]. Use of DEM and morphometric variables is shown as effective for ecological modeling [64], particularly forests mapping [65] and mammalian richness analysis [66]. We used 6 principal components and 4 topographic parameters in total.

Principal components are less suitable for SDM compared to spectral reflectance as we need to obtain response curves between model species and initial environmental variables. For SDM, we performed multicollinearity analysis to find which bands or indices are correlated. When the correlation coefficient of two bands or indices was greater than 0.7, one of them was removed. The following environmental variables were used for SDM—Landsat 8 June: band3, band4, band5, NDMI, NDVI; September: band3, band4, band5, NDMI, NDVI, and 4 topographic parameters.

Mosaic, principal components, and layer stacking analyses were carried out in ArcGIS 10.3 environment. Topographic coefficients were calculated in ENVI 5.0.

According to our field experience as well as many zoological studies [67,68,69,70]—ungulates can approach rather close to the villages and roads. Animals react to the infrastructure factor in different ways. Thus, we excluded these factors from analysis.

### 2.5. Map of Habitat Types

The assessment of spatial heterogeneity of habitats was performed in several stages. At the first stage, field data on tree species composition for each sample plot were classified by the *k*-means method [71]. *K*-means classification was carried out in SPSS 23 [72]. The resulting classes of forest ecosystems were supplemented by additional indicators: (i) tree stand density (forest/sparse forest), (ii) hydromorphic conditions (wetlands), and unsuitable habitats (farmland, settlements, water). Then, the obtained classes were used as training samples for modeling of the land cover spatial structure. Modeling was performed by supervised classification using the maximum likelihood classifier in ArcGIS 10.3 (ESRI, Redlands, CA, USA) [73]. A map of habitat types is presented as a mosaic of forest and non-forest patches belonging to different classes (habitat types).

### 2.6. Species Distribution Modeling for Tiger Prey Species

SDM for ungulates was implemented by two methods: (a) the maximum entropy method in MaxEnt software [74] and (b) linear discriminant analysis (LDA) in SPSS software [18,75]. Both methods were applied for each of the three tiger prey base species, including the wild boar, the roe deer, and the red deer. These two methods are different, and both are suitable for species distribution modeling [76]. These methods use different input data (training sample). The maximum entropy method uses presence data (GPS coordinates of footprints) and it generates random background points (pseudo-absences). The discriminant analysis uses both presence and absence (track points coordinates where no footprints registered). Morevoer, methods differ by the functions used. MaxEnt uses nonlinear (quadratic, product, hinge, and threshold) functions of environmental variables, as well as linear. It gives more flexibility but must be explained in more complicated terms. Detailed comparative analysis of Maxent and discriminant analysis is given in Appendix A.

#### 2.6.1. Integrated Map of Habitat Suitability Index and Comparison of MaxEnt and LDA Results

The map of the integrated suitability index (HSI) for the Amur tiger was produced by weighted summarizing of the HSI of three tiger prey species of wild ungulates. Weights are based on literature data obtained and described during the special case study of the tiger diet analysis [77]. The percentage of biomass in the tiger diet, according to the above study consisted of the wild boar—36.6%, the roe deer—11.45% and the red deer—4.19%. HSI integration was performed both for MaxEnt and LDA results. The comparison of MaxEnt and LDA results was performed by cross-tabulation.

#### 2.6.2. Habitat Ranking

We used spatial cross-tabulation of two raster maps to rank habitat suitability for three species of ungulates: the map of habitat types and the integrated map of habitat suitability. To rank habitat suitability for different land cover and land use types, the average weighted HSI was evaluated for each of the 17 habitat types (see in Results). For each type of habitat, the average value of suitability was evaluated. Habitat types were then ranked into 4 groups: (i) highly suitable, (ii) moderately suitable, (iii) low suitability, and (iv) unsuitable. Results of habitat ranking were grouped from unsuitable to highly suitable (0.3, 0.3–0.4, 0.4–0.6, >0.6, respectively). The total number of highly suitable forest patches was 90 616. the spatial fragmentation analysis was applied to a set of highly suitable forest patches to select the core areas of the potential ecological network.

### 2.7. Ecological Network

The Lesser Khingan land cover is heterogeneous, both in terms of habitat type and fragmentation level. The modern spatial structure of habitat types was formed under climate differentiation and human activity factors, including historical processes of land use. The northern part of the Lesser Khingan significantly differs from the southern part. Coniferous silviculture was typical for the northern part of Lesser Khingan due to extensive forest management in the past. Although the area of forest habitats is large in the northern part of the study area, it was less suitable for ungulates. In contrast, the broadleaf forests of the southern part were more suitable for ungulates and also covered a significant area. However, farming here negatively influences the habitat suitability level. Human-made infrastructure in the southern part of Lesser Khingan decreases the suitability of habitat for the wildlife. A set of various fragmentation metrics was used in order to take all these factors into consideration.

Spatial fragmentation metrics were calculated for a group of highly suitable patches (90,616). The patches with the least fragmentation (best integrity) were assigned as the cores of a potential ecological network. By least cost distance analysis, the corridors between cores were obtained. Finally, the current network of protected areas was audited, regarding its feasibility for sustainable tiger prey population and further tiger migration potential.

#### 2.7.1. Fragmentation Analysis and Spatial Structure of Ecological Network

There are at least three possible levels of fragmentation measurement—patch, class, and landscape level. In the current study we measured fragmentation at the patch level as well as we considered that fragmentation conditions of individual patches are more important to individual animals, both tiger and ungulates [78]. Nine spatial ecological metrics were calculated to estimate the fragmentation of patches for the focal group of the highly suitable habitats: area and perimeter, shape, core area, contiguity, proximity, similarity, Euclidean nearest neighbor (ENN), and edge contrast. We used nine different metrics for the balanced consideration of different aspects of fragmentation. A detailed description of the fragmentation metrics is given in (Appendix A). Fragstats 4.2 (US Department of Agriculture, Forest Service, Pacific Northwest Research Station: Washington, DC, USA) package was used [79].

A simple ranking approach was used considering the heterogeneity of habitat types and fragmentation of the Lesser Khingan. This approach allowed us to select the least fragmented patches within different sub-regions of the Lesser Khingan. For every fragmentation metric, the higher 0.1 percentile of patches was selected, which equaled approximately 100 patches. As soon as these nine samples are partially overlapping, the total quantity equaled 547 least fragmented patches of highly suitable habitats. These patches are considered herein after as cores of the potential ecological network.

#### 2.7.2. Ecological Corridors

The ecological corridors were designed by the least cost distance method [80,81,82], which uses the cost raster of animal migration factors. High cost indicates a more complicated (expensive) migration path, also called the friction of environment. The cost raster included three factors: closeness to settlements (meters), closeness to roads (active settlements and trafficked roads were selected and used to produce the cost raster), and the root mean square error (RMS) of elevation. The RMS of elevation means complexity of relief. Flat relief is easier to move through, whereas rugged terrain complicates movement of animals. Flat areas (both lowlands and uplands) had low RMS. Slopes, inflections, and rock outcrops had a high value of RMS. Sensitivity to terrain ruggedness during migration is especially typical for Amur tiger [24]. Three factors were normalized and summarized to the integral cost factor raster. The least cost distance method calculates the minimum cost path between neighboring cores. Cores and paths were then converted into graphs, and a minimum spanning tree was determined, which connects all cores by the minimum cost paths. Estimated corridors and cores become a structure of a potential ecological network for animal inhabitation and migration. All calculations were performed in ArcMap software.

## 3. Results

### 3.1. Habitat Types

Ten types of forest habitats were obtained as a result of the classification of tree species composition (Table 3): broadleaf dominated forests (## 1, 2 and 8); small-leaf forests (## 3, 4 and 10), conifers (## 5, 6 and 7), and mixed spruce with birch (#9).

**Figure 2:**
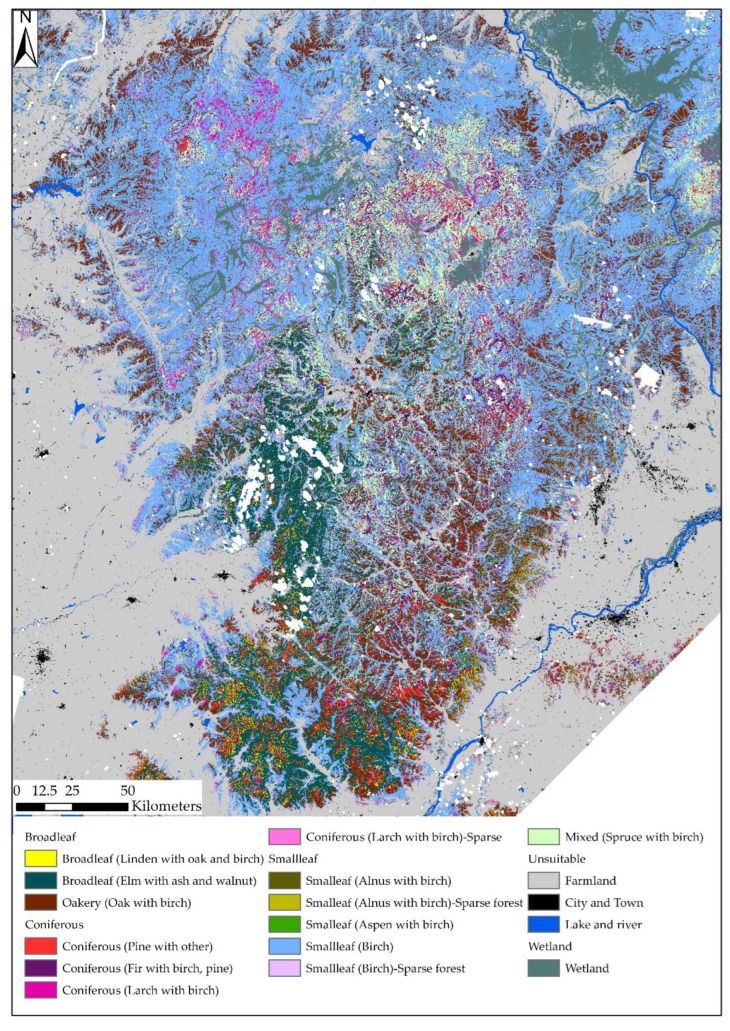
Map of habitat types of the Lesser Khingan obtained by supervised classification of environmental variables. Scale 1:1,500,000. White color corresponds to clouds and shadows in a multispectral image.

We added learning samples based on field observations for four non-forest classes: agricultural lands, wetlands, settlements, and water bodies, and we also added learning samples for three types of sparse forest: coniferous (larch and birch), small-leaf (alnus with birch) and small-leaf (birch). In total, 17 classes of land cover/land use were used for supervised classification and mapping. The result of mapping the spatial structure of habitat types is given in Figure 2. The map shows the effect of long-term anthropogenic impact. Most parts of the Lesser Khingan area are covered by secondary birch and sparse forests. This can be clearly seen in the north part of the Lesser Khingan. Secondary mixed forests and wetlands also occur in the central zone of the Lesser Khingan. The proportion of broadleaved species increases from 48° N to the south.

In the north section of the Lesser Khingan mountains, the vegetation distribution was formed by pine broadleaved and oak or birch-oak forests and then changed to oak-larch forest under anthropogenic impact. Moreover, wetland areas are presented in the north section of the Lesser Khingan mountains. The pine broadleaved forest was the dominant forest in the south of the Lesser Khingan mountains. Destroyed by clearcutting practices (years 1990–2000 according to field observations), this forest was reestablished as a broadleaf mixed forest.

### 3.2. Species Distrubution Modeling for Prey Species and Integrated Habitat Suitability Index for the Tiger

#### 3.2.1. MaxEnt

For Maxent model validation, we used several metrics such as training/test area under curve (AUC) and AUC difference. Model validation results are shown in Table 4, and are mirrored by the receiver operating characteristic (ROC) given by the MaxEnt model for the three main prey species of tiger. The AUC (Area Under the ROC Curve) values were close to or more than 0.9, which indicates good quality for all three models. Model overfitting evaluated by AUCdiff shows that the metrics are almost close to 0 which indicates low probability of overfitting.

##### The Relationship between Habitat Suitability and Environmental Variables

The contribution rates of each environmental variable output by the model are shown in Table 5. NDVI in June was the main contribution variable for three prey species. Elevation had low contribution rate to the roe deer and the red deer. Slope steepness had a low contribution rate to the wild boar and the red deer.

**Figure 3:**
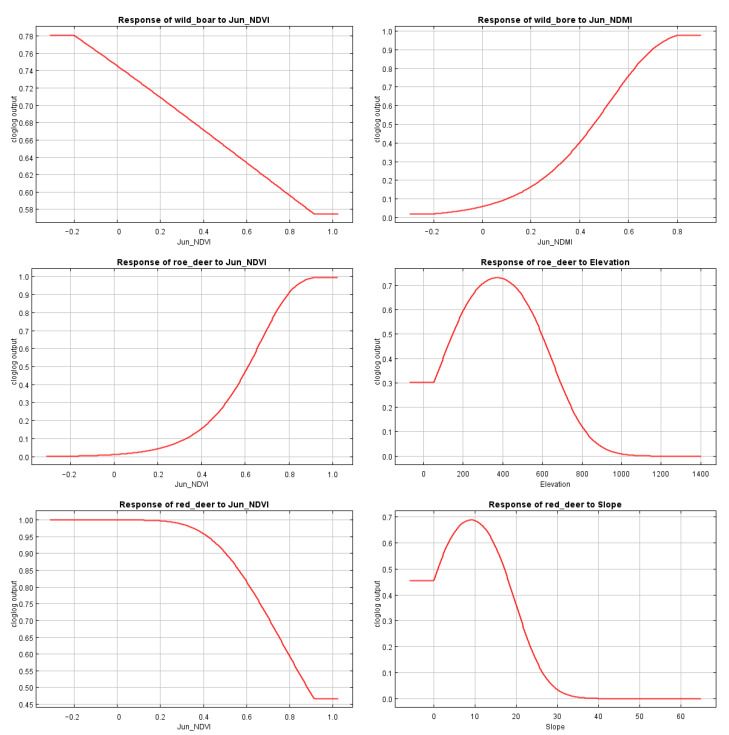
Response curves of three ungulates model prediction to environmental variables. Cloglog is the default model output, which is the simplest to understand: it gives a probability of species occurrence estimate between 0 and 1.

When modeling the three ungulates using the jackknife test, the important variables turned out to be band 4, NDVI, and NDMI in June, plus elevation and slope. It can be seen from the response curve (Figure 3) that the habitat suitability of the wild boar decreased with the increase in NDVI in June, but the curve did not enter the unsuitable range, indicating that the vegetation condition for wild boar is important factor but not a limiting one. In contrast, habitat suitability of the wild boar correlated with the increase of June NDMI, indicating that wild boar is mainly adapted to the habitat with high moisture content and dense vegetation coverage. The habitat suitability for the roe deer increased with the increasing June NDVI, which showed that quality of vegetation conditions play an important role in the roe deer habitat. The altitude response curve showed that the roe deer prefer the habitat in the range of 100–650 m altitude and the higher altitude area was not suitable for them. That indicates the important role of altitude as the limiting factor for the roe deer distribution. The habitat suitability of red deer decreased with the increase of June NDVI, indicating that red deer avoided dense vegetation canopy. Red deer optimal suitable habitat was calculated for 0–20° slope and the higher slope reduced habitat suitability.

##### Habitat Distribution Characteristics

**Figure 4:**
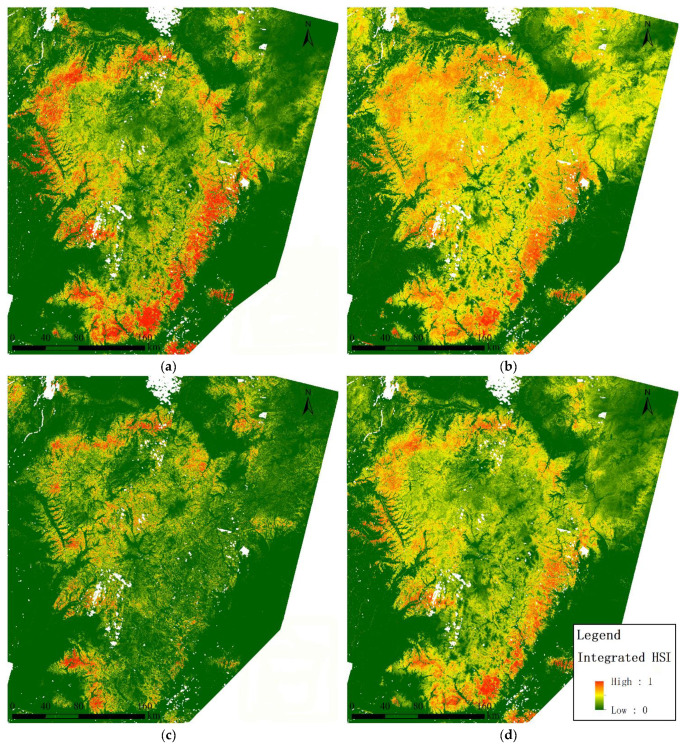
Prediction of potential habitats using MaxEnt modeling for (**a**) wild boar, (**b**) roe deer, (**c**) red deer, and (**d**) an integrated habitat map for the three tiger prey species.

Habitat suitability modeling and mapping was carried out (Figure 4) for the wild boar, roe deer, and red deer. Habitats with good suitability for the wild boar were distributed on the edges of the Lesser Khingan mountains and characterized with broadleaved forest and oak forest. Habitats of good suitability for the roe deer were distributed throughout the Lesser Khingan mountains, except for high altitude areas. Habitats of good suitability for the red deer were distributed in the north, west, and southern part of the Lesser Khingan mountains and were characterized mainly with broadleaved forests.

According to the integrated map of the suitability of tiger habitats based on the tiger prey’s biomass proportion distribution forecast (Figure 4d), the potential prey-based habitat of the Amur tiger is distributed on the edges of the Lesser Khingan mountains. In total, it is similar to the suitability habitat of the wild boar distribution, the large biomass proportion of wild boar in the food composition of the Amur tiger. A visual analysis of the integrated suitability map of tiger habitats revealed several features. The summarized area of sites with the highest suitability (>0.8) was extremely small. The most suitable sites were distributed throughout the Lesser Khingan with a slight predominance in the southern and eastern parts. Frequently, they were confined to the lower parts of the slopes of the valleys adjacent to the floodplains. In particular, the area adjacent to the border between China and the Russian Federation was one of such clusters of highly suitable habitats that can serve as a transboundary migration corridor. Highly productive oak forests with mesophytic shrubs and grasses are situated there.

#### 3.2.2. Linear Discriminant Analysis

**Figure 5:**
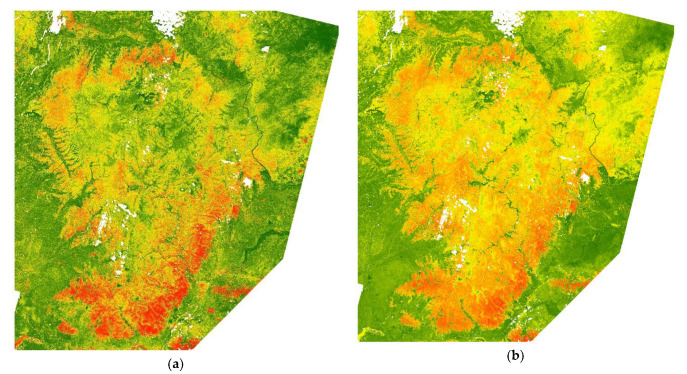

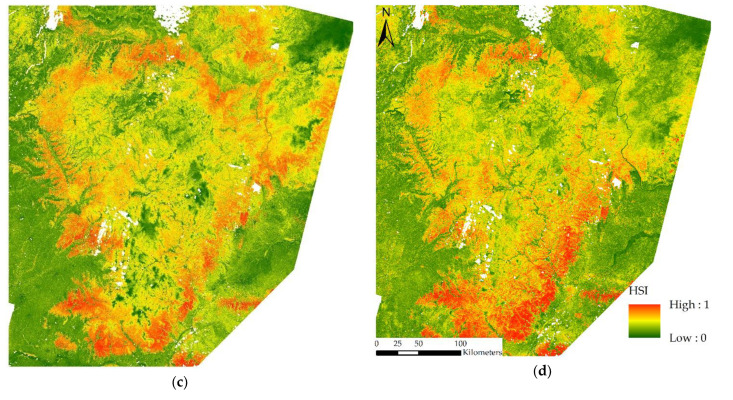
Habitat suitability index (HSI) for 3 species of ungulates according to linear discriminant analysis (LDA): (**a**) wild boar, (**b**) roe deer, (**c**) red deer, and (**d**) integrated suitability index.

Based on the same environmental dataset (Table 2), we also modeled the habitat suitability of ungulates by linear discriminant analysis. Table 6 indicates the main parameters of discriminant models for each prey ungulate, including the percentage of the correct discrimination of the “presence” state, according to the initial sample and the chi-squared test with degrees of freedom (*p* < 0.001). Amongst prey species, the boar was characterized by the highest percentage of correct discrimination (77.1%; Figure 5a). However, 12 of the 20 environmental variables were included in the model. The roe deer habitats correct recognition equaled 61.9% based on nine environmental variables (Figure 5b). Red deer habitats with less quantity of registration points had a 69.7% correct discrimination with only five environmental variables (Figure 5c). Variables included in the models are provided ©n Table 7.

Despite the highest number of roe deer footprint registration points, the roe deer model had the lowest quality of discrimination. Despite the highest quality of model for wild boar habitats, a large number of input environmental variables also influenced it, which signifies the “combinatorial” nature of the model. As more environmental variables are included in the model, more combinations could interact with each other and produce a higher level of discrimination. Accordingly, the predicted variable receives the higher mean of the statistical probability and possibilities of correct discriminations. However, not all of the variables included in the model had an interpretable physical interaction with the predicted variable.

The standardized coefficients of the environmental variables included in the model reflect the contribution of the variable to the discriminating functions (virtual variables, in the field of which the states of the predicted variable are best separated)—Table 7. The absolute value reflects the degree of contribution to the function, and the sign represents the type of connection (direct/inverse) with the value of the predicted variable. The habitats of all modeled species were characterized by a high correlation with spectral reflectance values in the Landsat bands 4 and 5 in June. Moreover, with a positive sign in band 4 and negative in band 5, the ratio of these bands is the basis for calculating the vegetation index and its variants (NDVI). When reflectance in band 4 increases, the reflection in band 5 decreases. NDVI along with net primary production are also increasing, respectively. Thus, the higher biological productivity (NDVI) in summer indicates the better habitat suitability for the modeled species. Reflectance in bands 6 and 7 (humidity) in June had a weak effect on the distribution of species habitats. The remaining vegetation indices in June had a multidirectional effect. For all prey species, the largest contribution was made by MSAVI, which reflects biological productivity (which should be adjusted when a large percentage of open soil surface presents). At the same time, for the wild boar and roe deer, the EVI index had a significant negative effect, which reflects the differentiation of productivity adjusted for the tree canopy (correct for some atmospheric conditions and canopy background noise and it is more sensitive in dense vegetation areas). This means that in June, the attractiveness of semi-open habitats increased (for all prey species) and the dense highly-enclosed forests decreased (for wild boar and roe deer). The leading determining factor for the suitability for wild boar and roe deer habitats sharply changes in September. In June, their main important factor was biological productivity; in September, there was the humidity—these factors were expressed through the NBR2 index and reflection in band 6. The reflectance was higher with lower humidity, i.e., the drier habitat is more suitable for the wild boar and roe deer. For wild boar habitats, this effect was also supported by a negative connection of wild boar HSI with the NDMI humidity index. For the wild boar and red deer, the habitat suitability in September was also positively related to biological productivity, expressed through NDVI and MSAVI indices. The latter was negatively related to the suitability of habitats, probably because it reflects the productivity of territories with small biomass (for the study area—there are anthropogenic areas in September, that include agricultural landscapes and settlements). A significant contribution to the model suitability was made by the elevation; the higher the elevation, the less suitable it is. For the suitability of wild boar habitats, slope also mattered; the steeper the slope, the more attractive the habitat.

According to the obtained models, the following can be concluded for the studied prey species: the main factors of habitat preference—in summer were heat supply and biological productivity, and the habitat preference in autumn was dependent mainly from the moisture factor.

Characteristics of sites with medium suitability for ungulates (0.6–0.8) were distributed evenly according to the integrated map (Figure 5d, color orange). In the northern part of the Lesser Khingan, they were typical for the middle and upper parts of the macroslopes with eastern and southern valley expositions (“sun spots”), low-mountain and mid-mountain watersheds with an elevation up to 400, less often up to 500 m. In the southern part of the Lesser Khingan, physical and geographical conditions were not clearly expressed. Forests with broadleaved species presence were mostly identified here. Areas with suitability from 0.4 to 0.6 (Figure 5 color yellow) were developed under the same landscape rules; the difference was in the composition of the forest where small-leaved species are in greater proportions. Floodplains and bottoms of river valleys were also described as habitats of medium suitability (except agricultural areas). Forested areas with medium suitability (0.4–0.8) were found to be extremely important for animals. Throughout the Lesser Khingan, they surround highly suitable habitats and as a result, they could be accepted as powerful ecological filters and buffers that reduce the factors of visual and acoustic disturbance from roads and settlements; they also neutralize air pollution and serve as a natural obstacle to the penetration of human activity and machinery.

Habitats in the southern part of the Lesser Khingan had low availability for all ungulates species due to two factors: agricultural intensity and midland elevations (>550 m.a.s.l.). In the northern part, habitats less suitable were wetlands and low-productivity forests (i.e., birch, spruce, fir forests, and light forests).

#### 3.2.3. Comparing MaxEnt and LDA Models

**Figure 6:**
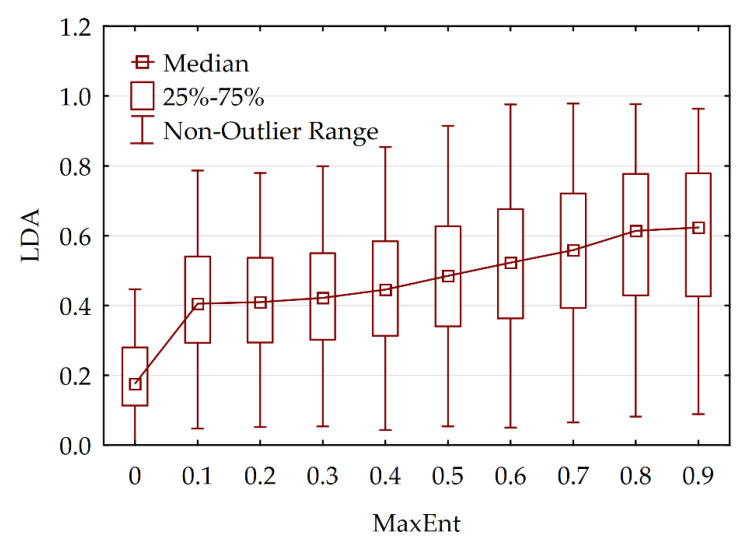
Box–whisker plot of cross-tabulation between MaxEnt and Linear Discriminant Analysis LDA integrated models.

We overlaid and cross-tabulated two maps of the integrated habitat suitability index: MaxEnt map (Figure 4d) and LDA map (Figure 5d). The results show that, in general, the two models were linearly correlated. However, the MaxEnt model shows more diversity, which can be interpreted as more sensitivity to environmental variables (Figure 6).

Both methods demonstrate homomorphic habitat suitability maps (Figure 4d and Figure 5d). Thus, in accordance with both models, the highly suitable habitats occupy the southern and southeastern outskirts of the Lesser Khingan, as well as the wide stripe of habitats along the right bank of the Amur river valley.

#### 3.2.4. Habitat Ranking

Broadleaf forests, with the highest suitability, averaged 0.63 HSI and occupied almost 2 million hectares; they are distributed primarily in the southern part of Lesser Khingan. Birch (with minor mixed forests) with moderate suitability (0.42 HSI) had the largest area with more than 4 million hectares in the central and northern Lesser Khingan. Mixed and coniferous forests with average suitability value of 0.32 HSI occupied 1.2 million hectares (Table 8).

### 3.3. Fragmentation and Ecological Network

Large patches of broadleaf forests in the southern part of Lesser Khingan have high values of functional metrics—area and perimeter. On the contrary, forest patches in the central and northern Lesser Khingan have high values of functional and connectivity metrics, such as similarity and edge contrast. This effect can be explained by the buffer role of less-suitable coniferous and mixed forests that surround broadleaf forest patches as buffer zones. These forests support a relatively homogenous environment for ungulate prey species in Northern part of Lesser Khingan. In contrast, for the southern part of Lesser Khingan, the huge territories of broadleaf forest patches are surrounded by farmlands with numerous roads. Therefore, nine different fragmentation metrics were used for balanced consideration of various aspects of fragmentation. The values of fragmentation metrics for 547 patches are provided in Table A5. These patches are considered potential cores of an ecological network.

#### Analysis of a Potential Ecological Network; How It Coincides with Existing PA

**Figure 7:**
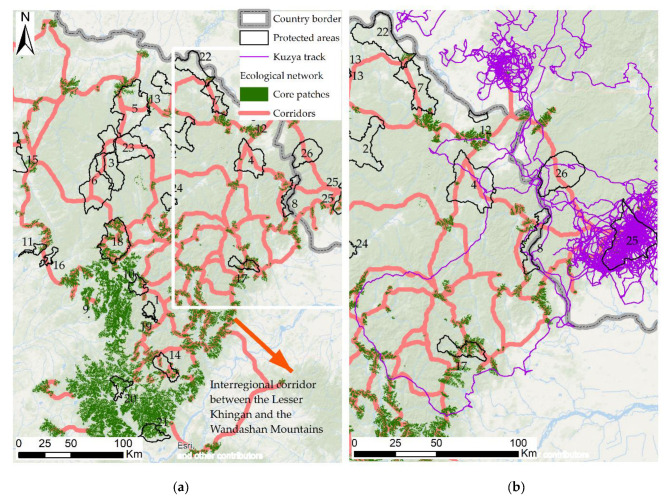
(**a**) Potential ecological network of the Lesser Khingan; (**b**) fragmentation of potential ecological network along the Amur River valley overlaid with track of tiger Kuzya [3]. Protected areas: 1—Liangshui, 2—Wuyiling, 3—Youhao, 4—Xinqing, 5—Hongxing, 6—Dazhanhe, 7—Maolangou, 8—Taipinggou, 9—Shuangbaoshan, 10—Wumahe, 11—Zhayinhe, 12—Jiayin, 13—Duerbinhe, 14—Langxiang, 15—Shankou, 16—Shuangchahe, 17—Xinlinhe, 18—Heyuantou, 19—Bishui, 20—Pingdingshan, 21—Longkou, 22—Pingyanghe, 23—Kuerbinhe, 24—Fenglin, 25—Lichun, 26—Zhuravliny.

The potential core patches were used to build a corridor network by the method of least cost distance. Cores and corridors calculated for the study area are shown in Figure 7a, along with the current network of Chinese and Russian protected areas.

Animals confirmedly use the designed corridors and predicted transit ways in real time. That was confirmed by GPS tracking from the collar of a male tiger, Kuzya, reintroduced in the Russian Federation in May 2014. Kuzya crossed the border from Russia to China during the process of home range establishment in its southern part and migrated over the Lesser Khingan in 2014 [3]. The tiger movement indicated by GPS tracking partially coincided with the modeled corridors and follows along and near them (Figure 7b). The tiger does not avoid proximity to settlements, but it also does not converge closer than 500–1000 m. It also can be assumed that designed corridors may be indicated as key directions of wider migration belts.

Most ecological cores intersected by Kuzya’s track [3] are located in the east part of Lesser Khingan. We suppose that this area might be preferable for animals during their spread, migration, and investigation of their potential home range territory than for permanent use. These types of activity were detected during the period when this tiger-male visited China. It also could be seen that native Russian potential core patches are also attractive for the tiger but with the reason that they need to be surrounded with significant forest buffer zones of 10–15 km from the outer border.

## 4. Discussion

### 4.1. Identification of Potentially Suitable Habitats for the Tiger in the Lesser Khingan Mountains

**Figure A1:**
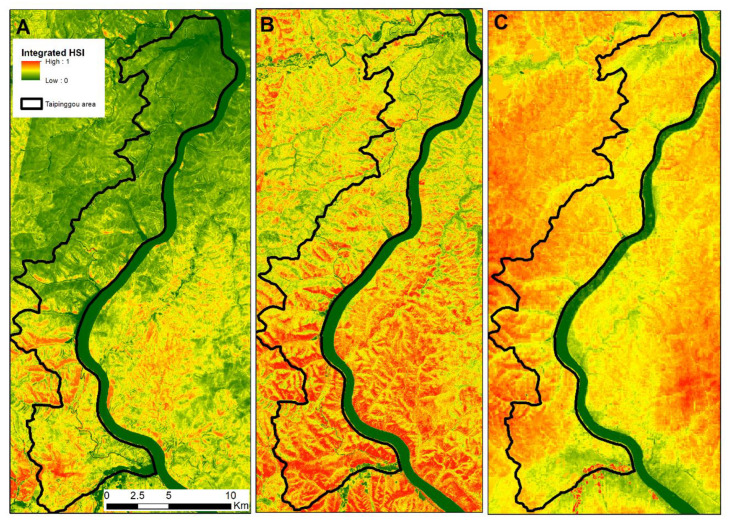
Three integrated prey-based habitat suitability models: (**A**) MaxEnt modeling based on the Lesser Khingan winter track census survey, (**B**) discriminant analysis based on the Lesser Khingan winter track census survey, (**C**) discriminant analysis based on Taippingou winter track census survey.

**Figure A2:**
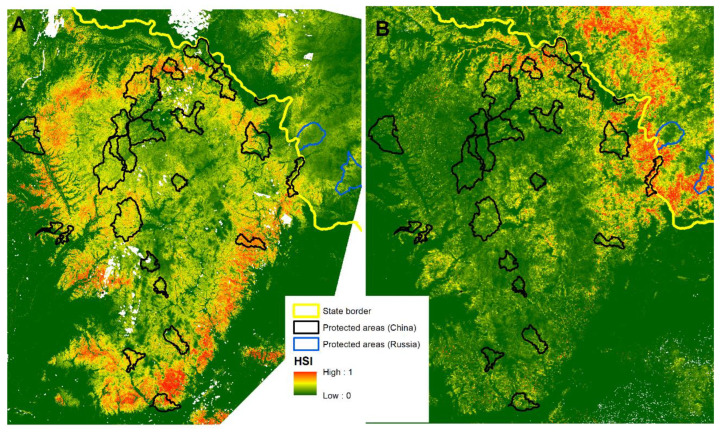
(**A**) Integrated prey-based MaxEnt model based on the Lesser Khingan winter track census survey, (**B**) MaxEnt model based on data from GPS-collars on tigers.

Our work concentrated on the habitats in the Amur tiger historical range—an area that was empty with tigers during the last years. Here, estimated habitats of the tiger prey and our results on the northern and northeastern spurs of the Lesser Khingan partially coincided with the habitat suitability model developed earlier for the tiger only and were based on GPS data from tiger collars [83] (Appendix A, Figure A1, Figure A2). Additionally, our results partially coincided in the Taipingou National Park and its environs. All these confirm that distribution of described Lesser Khingan habitat types (Oakery, broadleaf, small-leaf forests) has principal importance for the potential recovery of tiger and its prey-base.

Thus, the ecological region and its habitats may need priority in the protection measures both for tiger and its prey base. Historically, the two territories (modern Russian and Chinese) we are talking about here were under different types of forest management. The forests in Russia are mostly mature and overmature (lack of proper forest management due to remote location from weak infrastructure), while the forests in China were until recently under the high pressure of extensive development. Despite that, existing vegetation cover of the north-eastern Lesser Khingan and the adjacent area of the Russian Federation still represent a natural way for transboundary movings of tigers and prey base species. This is also true for the Taipingou National Park.

### 4.2. Analysis of Fragmentation and Potentially Suitable Ecological Cores and Corridors

The mosaic structure and fragmentation of habitats were revealed for the Lesser Khingan mountains with the current study. Our models predict that the suitability gradient increased with the proportion of broadleaved tree species in the tree species composition, and with the productivity of vegetation. This lay in the same field of results and has been received for different areas of the Amur tiger range [38,46,84] and for the same study area, but with other methods [18,83]; it also corresponds to the results of the study on Taipingou National Park [18]. Here, we could also note that regional variation in the distribution of biotopes (in terms of their productivity) stands out against the trend for the whole region. That should be taken in account during conservational planning and biotopes’ recovery.

When discussing the habitat features of corridors, we have found that movements along sloping surfaces are typical for both animals (tiger and prey) [80], regardless of the season. Investigations based on GPS data from the collars of wild tigers in the region of Russian Far East Primorye [79] confirm the results of current study, that the deciduous and broadleaved forest formations as suitable habitats for the tiger and its prey are extremely important. Habitat suitability index for group 1 is highest (Table 7)—that means that habitats of that group are the best, and are most suitable for tiger prey. As natural corridors are used by animals for moving, they prefer ecotone habitats which are mainly located at the forests’ edges and characterized with various canopy structures and tree species composition [80].

**Figure A3:**
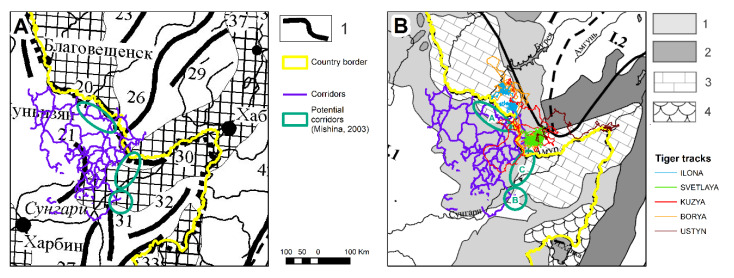
Combining the results and regional zoning schemes. (**A**) Orographic diagram of the basin of Amur River (Heilongjiang) [55] and potential corridors calculated using the least cost method. Symbols on the map A: 1—mountain ranges and systems of ridges, 2—plains. (**B**) Floristic areas of Northeast China [85] and tiger GPS tracks (lines of different colors marked with the tigers’ names Ilona, Svetlaya, Kuzya, Borya, Ustin [3]); designations on map B: 1—Manchurian mixed forests, 2—Ussuri broadleaved and mixed forests, 3—Amur wetland and wet steppes, 4—Ussuri wetlands and forest-steppes. Green circles indicate regions with potentially important green corridors of level 1.

Wild tigers prefer habitat borders and areas of extensive coniferous forests for far distance migrations; simultaneously, as shown here when calculating ecological corridors. To discuss the “corridors” we calculated in the current investigation, we also compared our results with the well-known regional maps of the Russian and Chinese Far East. We have merged maps where the orographic scheme of the Amur River basin [55] is combined with potential corridors of the ecological network designed by us here (Figure A3) and floristic regions Figure A3Bof Northeast China [85]. Furthermore, we merged these maps with data received from GPS collars of five tigers that were reintroduced by us [3] (Figure A3).

Most ecological key areas were also intersected by the reintroduced tiger named Kuzya [3], as mentioned above. These key areas are located in the east part of Lesser Khingan. He also used the territory of the Taipingou National Park. We suppose that the Taipingou area might be preferable for animals during their spread, migration, and investigation of their potential home range territory than for permanent use. These types of activity were detected during the period when this tiger male visited China. It also could be seen that native Russian potential core patches are also attractive for the tiger but with the reason that they need to be surrounded with significant forest buffer zones of 10–15 km from the outer border.

Synthesizing all that has been mentioned, we obviously can say that tigers preferred migration paths lying along the ecotones of different plant formations, for example, mixed forests/wet steppes, or broadleaved mixed forests/wet steppes. Thus, the potential corridors estimated in the current study might be considered as regional (small scale) green corridors for tigers. The large forest/steppe ecotone might be considered a transboundary (large scale) migration corridor. The transboundary corridors (Figure A3) included ***Corridor A***—the foothills and low mountains of the northeastern and northern Lesser Khingan (right side of the Amur (Heilongjiang) river valley and Songhua River valley). The corridor elongated from the southeast and it can probably extend further in northwest direction toward the Greater Khingan mountain system; ***Corridor B*** is the important connection between the Lesser Khingan and the western part of the Wandashan mountain system. It also went further to the southeast directly to Khanka Lake (Sinkaikhu Lake); and ***Corridor C*** is organized by the foothills and low mountains of the eastern part of Lesser Khingan.

It seems to be the best way to restore territories under the ecological paths and corridors—to plant native conifers (the fastest growing) species—pine, larch, fir, and spruce. This planting activity will reduce the degree of tiger habitat fragmentation in the Lesser Khingan. Native conifer tree species are characterized with a relatively fast growth rate, what is necessary for canopy closure and the understorey shading [86]. The planting of native conifers should be followed by thinning and tending cuts; it helps in the fast accumulation of tree biomass. The introduction of broadleaved species of trees into silviculture is necessary to increase plant biodiversity and provides the basis for a nemoral, mesotrophic, and a broadleaved grasses ground layer. A combination of multi-storey and multi species forest stands will provide a sustainable habitat for the tiger prey base. Implementation of silvicultural practices will also reduce the habitat fragmentation within the Lesser Khingan. Such methods being used will reduce fragmentation to approach the target indicators of the patch area, which was also proposed by Hebblewite et al. [38,84] for the territories of the Changbaishan mountains (the modern southeast range of the Amur tiger).

**Figure A4:**
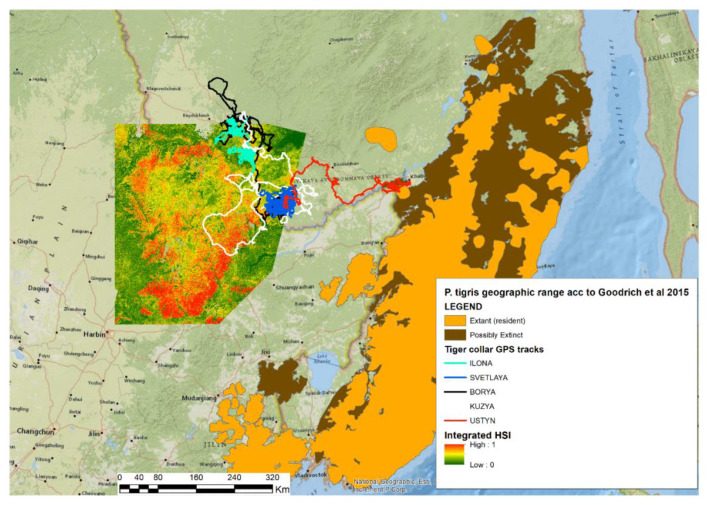
Tiger geographic range [1], integrated with our resulting prey-based HSI model and with pure GPS tracks received from reintroduced tigers’ collars.

**Figure A5:**
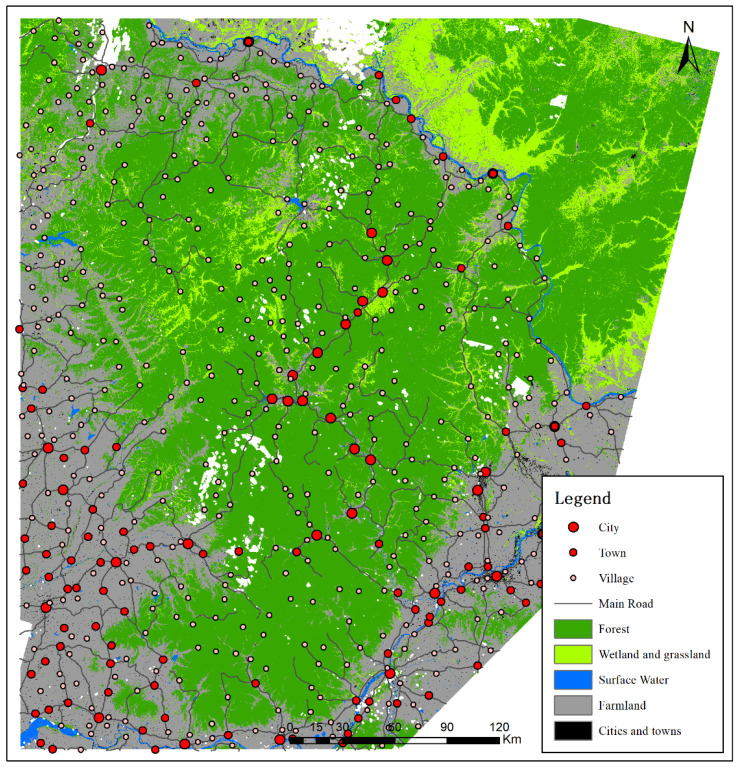
Map of human disturbance and anthropogenic activity of Lesser Khingan.

The discussion of the habitat range is really relevant [1] and our studies indicate that the conventional range of the Amur tiger is artificially narrowed (Figure A4).

### 4.3. Analysis of the Current System of Protected Areas and Network of the Tiger Key Territories

More than 90% of the calculated tiger key areas (cores of the ecological network) are located in the southern part of Lesser Khingan. However, this part of the study range is almost not supported by the protected areas. Only five from a total of 24 protected areas are located here, including Wumahe, Liangshui, Langxiang, Pingdingshan, and Longkou. The inner part of Lesser Khingan is characterized by the structure of complicated migration routes. It was less impacted by human infrastructure and has a good network of protected areas (Youhao, Hongxing, Dazhanhe, Kuerbinhe, Fenglin, Heyuantou). North and northeast spurs of Lesser Khingan have a rather dense network of ecological corridors, which are also well supported by a network of protected areas, including Pingyanghe, Maolangou, Jiayin, Wuyiling, Xinqing, and Taippingou.

The wildlife protection work in the territories adjusted to the Taippingou protected area as an important location for a transboundary migration route of tigers between Russia and China (***Corridor A***) should be developed. Several border crossings by tigers are registered here [3] regularly. Interregional connection in the southeast of the study area described above (***Corridor B***) has a sustainable tiger population, and is well monitored in the Wandashan mountains. This corridor should be supported with biotechnical measures and acquire status of strong protection as an important regional tiger migration path. Now it has no status of protected area.

The connection of the Lesser Khingan with the Greater Khingan mountains is by a few poor and structurally complicated corridors registered at the northwest of the study area and it is also not supported with protected areas. Only one connection with the Zhangguangcailing mountains exists at the southern part of Lesser Khingan, which could be developed as a potentially protected ecological corridor. It has no protected status nowadays. To support this territory and protect it, it will allow tigers of the Lesser Khingan to move to the tiger population area inhabiting the “National Park for Amur tigers and Amur leopards” in China.

Strategies, action plans, and special programs designed to preserve the tiger provide activities to restore the species and its range. We strongly recommend using the results of our scientific investigations, including the development of cross-border activities for wild nature protection.

Conservation measures could be implemented both in Russia and China, within the natural boundaries of the tigers’ distribution, regardless of governmental borders. Most of the mountain system of the Lesser Khingan is situated inside the area of the tiger current range. The restoration of this species here is necessary and justified. That is confirmed with both types of habitat modeling results presented in this paper, and could be argued. The success of the tigers’ reintroduction in the Amurskaya region, Khabarovsk region, and the Evreyskaya autonomous region (Russia) [3] and moreover, the historical distribution of the tigers [83], also supports that. Before implementing any reintroduction measures, it is necessary to connect ecological corridors into a single net-system of suitable habitat areas.

### 4.4. Discussion of methodological features

The approach of our study for fragmentation estimation and corridor calculation was partly similar in methodology and results to one by Hebblewhite et al. [84] in the Changbaishan mountain region. However, these two study areas do not intersect; and the core areas in Changbaishan were described as a larger area than those in Lesser Khingan. Our model was more sensitive to factors of fragmentation and had a less generalized result. Hebblewhite et al. [84] used a landcover map with four key vegetation communities, net primary productivity, and the percent of snow cover. The least cost method was implemented in a rather similar way in both studies. A study by Santos [87] aimed to determine the most significant forest patches and build corridors between them. As in our case, the significance of patches was based on multiple fragmentation metrics. Patches with the best metrics were selected by fuzzy logic algorithm. Contrary to our study, the cost raster was generated from fragmentation metrics. Protected areas inside the corridors were detected to be in misuse and not complying with environmental legislation. In total, our results describe a similar situation for different area of tiger range. The protected area network needs to be reorganized or complemented with new areas for the most effective use of the territory for tiger recovery.

## 5. Conclusions

In the current study, we implemented means of GIS spatial analysis into the potential habitats of the Amur tiger in Lesser Khingan. The proposed combination of data and methods can be used for decision support during the planning of binational transboundary protection programs for wild animals, both in Russia and China. Especially, it is relevant for the tiger and its prey.

The modeling of suitable habitats allows to (i) optimize the protected areas network [88,89] and design the transboundary status for protected areas of the Lesser Khingan; (ii) develop the basis for Biodiversity Action Plans in local areas of Heilongjiang province (PRC); (iii) restore the tiger population as an umbrella species for the whole diversity of native ecosystems and species [89,90]; (iv) identify potential corridors and design programs to improve the landscape and thus the wild key habitats’ area connectivity.

Our study concludes (1) the existence of natural guides and directions for tiger resettlement, (2) the existence and structure of tiger-relevant habitat types, and (3) that habitats are good for both the tiger and ungulates are associated with defined floristic complexes.

Restoration of tiger habitats will need a set of actions requiring coordination at the government and inter-government level, with a high responsibility on decisions and significant financial costs. Such actions are important now not only for the Amur tiger and North China [88,89] but also for the South Chinese tigers (*P. t. amoyensis*) and their habitats, because this subspecies has already disappeared from this region [91]. This is also relevant for significantly disturbed habitats of the Indochinese subspecies of the tiger (*P. t. corbetti*) [92].

The results of our work can be used for the future estimations of habitat fragmentation and further for ecological network calculation, which can be a scientific ground for a potential system of protected areas of the Lesser Khingan. The Amur tiger has all chances to be potentially recovered in North China, particularly in the Lesser Khingan. Russia–China transborder protection of the tiger habitat will be able to support a full-fledged Amur tiger population with the ability of young individuals to disperse and move in all directions of its historical range. Existing protected areas should be maintained by the special ecological network focused on Amur tiger protection. The current system of protected areas is rather dense and well developed; however, it is not aligned with the structure of suitable habitats for tiger prey species. The southern part of Lesser Khingan lacks protection measures. We consider that it is necessary to develop an inter-governmental Biodiversity Action Plan for the development of an ecological network in Lesser Khingan. This plan should include a set of biotechnical and forest management measures to restore and sustain ecological cores and corridors for tiger and its prey species, as well as other native ecosystems and species.

## Figures and Tables

**Table 1:** Example of field data collection.

GPS Location		Animal Information		Upper Canopy Layer	Subordinate Canopy Layer (s)	Understorey
Latitude	Longitude	Species	How Old Footprint Is			
46.2466517° N	129.0932033° E	Roe deer	1 day	3 Oak, 7 Birch	10 Aspen	10 Acer

**Table 2:** Animal census in sample plots (amount of data was collected in the field).

Year	Routes, km	Amount of Animal Footprints Points	Animal (Including Multiple Occurrences)			Sample Plots	
			Wild Boar	Roe Deer	Red Deer	Forest	Wetland. Farmland
2017	no data	162	2	94	12	99	63
2018	no data	405	218	356		315	90
2019	112.5	874	307	1708	112	858	16
**Total**		1441	527	2158	124	1272	169

**Table 3:** Composition of tree species in upper storey. Table represents results of k-means classification based on tree species composition. Columns represent 10 types of forest habitats. Numbers in cells are percentages of certain tree species in each forest habitat type. F and *p*-value demonstrate representativeness of each forest habitat type classification.

			Number of Habitat Type									
			1	2	3	4	5	6	7	8	9	10
	f	*p*-Level	Proportion of Tree Species in Upper Storey									
Oak	662.9	<0.005	16.2	2.3	8	9.5	6.3	0.7	5.9	79.9	1.5	2.1
Birch	392.9	<0.005	14.7	7.4	20.6	71.5	4.7	16.4	17.5	10.7	24.4	17.7
Pine	377.7	<0.005	5	7.3	1.5	0.9	51.7	13.8	0.2	1.1	4.7	0.6
Larch	580.8	<0.005	0.5	0.2	3.5	4.5	0.2	1.8	65.2	1.8	8.8	2.9
Alnus	539.8	<0.005	0.3	0.2	0.6	2.1	0	0.7	1.3	0.3	1.8	68.7
Aspen	282.3	<0.005	5.8	2.6	52.9	5.3	3.6	3.9	2.8	2.8	2.8	0.6
Fir	435.5	<0.005	0.8	0.8	0.6	0.6	6.4	48.3	0.2	0	3.7	1
Linden	253.6	<0.005	39.8	2.8	2	1.1	9.9	0.8	1	1.9	2	1.6
Maple	7.02	<0.005	5.6	3.6	3.9	1.5	4.5	1.9	0.9	0.5	1.5	1
Elm	84.3	<0.005	4.1	23.9	1.9	0.7	3.1	0.8	1.1	0.4	0.2	0
Spruce	361.9	<0.005	2	1.1	1.9	1.7	5.7	8.8	2.5	0.1	48.6	3.5
Ash	106.4	<0.005	3.5	23.5	1.6	0.5	2.3	1.1	0.7	0.3	0	0
Walnut	65.5	<0.005	0.2	14.6	0.8	0	1	0.6	0.4	0.2	0	0
Amur chokecherry	7.55	<0.005	0.1	2.6	0	0	0.1	0.3	0.1	0	0	0.3
Amur cork	25.3	<0.005	1.4	7.1	0.2	0.1	0.5	0.1	0.2	0	0	0

Color marked cells mean dark colored—dominant species, light colored—subdominant species. Habitat types: 1. Broadleaf (Linden with oak and birch), 2. Broadleaf (elm with ash and walnut), 3. Small-leaf (Aspen with birch), 4. Small-leaf (birch), 5. Coniferous (pine with other), 6. Coniferous (Fir with birch, pine), 7. Coniferous (Larch with birch), 8. Oakery (Oak with birch), 9. Mixed (Spruce with birch), 10. Small-leaf (alnus with birch)

**Table 4:** ROC curve (receiver operating characteristic curve) verification of three ungulates with MaxEnt prediction.

Parameter	Wild Boar	Roe Deer	Red Deer
Training AUC	0.902	0.811	0.911
Test AUC	0.875	0.804	0.820
AUCdiff	0.027	0.007	0.091
Regularized training gain	1.237	0.624	1.475
Unregularized training gain	1.431	0.713	1.718
Unregularized test gain	1.155	0.694	0.697
Standard deviation	0.012	0.008	0.043

**Table 5:** Percent contribution of environmental variables.

Wild Boar		Roe Deer		Red Deer	
Variable	Percent Contribution	Variable	Percent Contribution	Variable	Percent Contribution
June NDVI	33.1	June NDVI	68.8	June NDVI	49.9
June NDMI	25.8	Elevation	6.7	June band3	12.5
June band 4	7.1	June NDMI	4.7	Slope	9.3
Slope	6.8	June band3	4	June band4	8.4
September NDVI	5.5	June band5	3.7	Elevation	7.7

**Table 6:** Main discriminant analysis results for main prey species.

	Meeting Points Quantity	Percent of Correct Recognition	Chi-Square (df)
Wild Boar	309	77.1	815.98 (12)
Roe deer	814	61.9	724.58 (9)
Red deer	67	69.7	109.82 (5)

**Table 7:** Variables included in the discriminant model.

Variables	Standardized Canonical Discriminant Function Coefficients		
	Boar	Roe Deer	Red Deer
Spectral reflectance (June)			
Band 4	1.697	0.912	2.346
Band 5	−6.248	−4.461	−1.833
Band 6	0.073	-	-
Band 7	-	−0.616	-
EVI	−1.593	−2.429	-
MSAVI	9.863	8.234	3.717
Spectral reflectance (September)			
band 5	-	-	-
band 6	0.870	0.766	-
NBR2	−0.461	−0.229	-
NDMI	0.249	-	-
	-	-	-
SAVI	-	0.418	-
NDVI	3.557	-	-
MSAVI	−3.126	-	−1.676
Morphometrical parameters			
Elevation	−0.258	−0.270	−0.661
Slope	0.131	-	-

**Table 8:** Results of raster cross-tabulation of habitat types and habitat suitability index.

Habitat Type	Area, ha × 10^3^	Average HSI
**Group 1 (Broadleaf) highly suitable**		
Oakery (oak with birch)	1279.8	0.62
Broadleaf (elm with ash and walnut)	568.1	0.64
Broadleaf (linden with oak and birch)	84.8	0.64
Small-leaf (alnus with birch)	0.12	0.65
Total	1932.76	0.63
**Group 2 (Birch with larch) moderately suitable**		
Small-leaf (birch)	3888.6	0.42
Coniferous (larch with birch)	335.4	0.39
Coniferous (pine with other)	128.1	0.45
Smalleaf (aspen with birch)	45.2	0.45
Total	4397.4	0.42
**Group 3 (Spruce with birch and fir) low suitability**		
Mixed (spruce with birch)	926.2	0.31
Coniferous (fir with birch, pine)	330.5	0.35
Small-leaf (alnus with birch)—sparse forest	11.4	0.21
Small-leaf (birch)—sparse forest	1.63	0.21
Coniferous (larch with birch)—sparse forest	4.02	0.17
Total	1273.56	0.32
**Group 4 (unsuitable)**		
Farmland	4905.6	0.11
Wetland	1233.0	0.11
Water bodies	157.9	0.10
Populated areas	77.9	0.10
Total	6374.2	0.11

## Data Availability

The data presented in this study are available on request from the corresponding author. The data on GPS locations are not publicly available because access to them increases the vulnerability of wild animals and their accessibility for poachers (both rare tigers and ungulates). Remote sensing data (Landsat-8 and SRTM DEM) can be downloaded from https://earthexplorer.usgs.gov/, accessed on 1 September 2020. Human population density data can be downloaded from: https://zhuanlan.zhihu.com/p/448677461, accessed on 1 October 2022; cities and road data can be downloaded from: https://www.webmap.cn/commres.do?method=result25W, accessed on 1 October 2022.

## Appendix A

### Appendix A.1. Comparative Analysis of Maxent and Discriminant Analysis

#### Appendix A.1.1. Maxent

MaxEnt is a prediction model of potential species distribution which is based on the principle of an ecological niche [93]. The model constructs the environmental conditions of target species occurrence and is based on the environmental characteristic variables related to the “occurrence point” of species. The model interpolates the potential distribution to the whole area covered by environmental variables [74,94].

MaxEnt uses a receiver operating characteristic (ROC) curve for model quality evaluation based on test sample. For three ungulates, 25% of the occurrence points were set as a test sample and 75% of the data was set as a training sample. We used linear, quadratic, and product functions for environmental variables. Hinge and threshold functions were discarded as they were too complicated for interpretation. Replicate quantity was set 1 (type: cross-validate), output format—logistic. Regularization values: linear/quadratic/product: 0.050. The knife cutting method was selected to measure the weight of each variable and the response curve of each environmental variable was created. Other parameters are set by default.

The model overfitting was evaluated by minimum difference between training and test data (AUCdiff). This metric is based on the intuitive notion that overfit models should generally perform well on training data but poorly on test data [95]. By minimizing the difference between training and test data, we minimize the risk that our model is over-parameterized in such a way as to be overly specific to the training data.

#### Appendix A.1.2. Discriminant Analysis

The same environmental variables, as well as the same training sample (footprints registration coordinates—data of animal presence), complemented with the field researchers’ track points where no animal footprints were registered (data of animal absence). The LDA algorithm selects the discriminant function for the training sample that best separates the presence from absence of the species, depending on environmental variables. Then, the discriminant function is applied (interpolated) to the entire study area. The LDA produces for each raster pixel: (i) probabilities of “footprint presence” and “footprint absence” and (ii) value and quality of discriminant function. The positive region of discriminant function corresponds to trace presence probability.

Two options are available in LDA: (i) prior probabilities for each class estimated from group size and (ii) group probabilities for those considered equal. The first method gives the minimum probability of potential detection. For such extremely rare events as the animal’s footprint registration, the estimated probability is more realistic. Under this option, LDA predicted extremely limited areas of species presence (less than 1% of the study area). Therefore, the equal probability class was used in analysis.

### Appendix A.2. Fragmentation Metrics

Area and perimeter are the basic metrics, which indicate the inner capacity of each forest patch and its interaction with neighbor patches, also known as edge effects or ecotone capacity [96]. The core area metric is important if we consider the species does not use the whole area of the patch but rather its inner space, limited by some buffer distance between the core of the patch and its edge. This distance was defined in user-tuned matrix (Table A2). Core area metric is especially significant for edges between significantly heterogenous patches (i.e., forest/farmland), where the vegetation, microclimate, and noise factors may produce a buffer zone of 50–200 m, leading to the decrease of core area [97]. The edge contrast index (ECON) measures the degree of contrast between a patch and its neighborhood. Formula (1) is provided below. Weights of edge contrasts between the group of highly suitable habitats and other groups were established in a user-tuned matrix: a value of 0.2 indicated contrast to mid-suitable forests, a value of 0.5 had low suitability, and a value of 1 was assigned to contrast with unsuitable habitats. The contrast value between patches of highly suitable habitats was considered 0 (Table A3).

Another approach to evaluate edge effects is to measure the shape of the patch and its complexity [98]. Two metrics were used: shape and contiguity. Patch shape is a simple metric based on the ratio of area and perimeter of patch. Shape metric relates to the complexity of the patch shape in comparison to a standard shape (square) of the same size. Contiguity metric [99] is quantified by convolving a 3 × 3 pixel template with a binary digital image. A value of “1” was assigned to pixels within the patch under analysis and a “*zero*” value to the background pixels (all other patch types).

The Euclidean nearest neighbor (ENN) metric is the distance from the center of patch *i* to the center of the closest patch *j*, which belonged to the same class. The ENN metric has the disadvantage that closely located small patches may have improperly emphasized ecological value. To overcome this limitation, the proximity metric was used. For each patch, the size and distance to all neighboring patches of the same type (within some specified search distance) are enumerated. A patch with lots of other large patches in proximity will have a large value of proximity metric (i.e., low isolation). Thus, the proximity metric comprehensively considers both the distance between the patches and their area. Neighborhood was considered 10 km on the basis of daily migration potential data [24].

The similarity index is a modification of the proximity index, the difference being that similarity considers the size and proximity of all patches, regardless of class, whose edges are within a specified search radius of the focal patch. Specifically, the index distinguishes sparse distributions of small and insular habitat patches from configurations where the habitat forms a complex cluster of larger, hospitable (i.e., similar) patches. All other things being equal, a patch located in a neighborhood (defined by the search radius) was deemed more similar (i.e., containing greater area in patches with high similarity) than another patch will have a larger index value. The neighborhood was also considered 10 km. The similarity of focal patches was established in the user-tuned table (Table A4). A value of “1” was assigned to patches that were a highly suitable group, “0.8” to the moderately suitable group, “0.2” to the low suitability group, and “0” to unsuitable habitats. Metrics formulas provided in Appendix A (A1)–(A5).

EDGE CONTRAST = (∑^m^_k = 1_ *p_ijk_*d_ik_/p_ij_)*100, (A1)

p_ijk_—length (m) of patch edge ij adjacent to patch type (class) k, d_ik_—dissimilarity (edge contrast weight) between patch types i and k, p_ij_—length (m) of perimeter of patch ij.

SHAPE = (0.25*p_ij_)/√a_ij_, (A2)

p_ij_—perimeter (m) of patch ij, a_ij_—area (m^2^) of patch ij, 0.25 is a constant to adjust for a square standard.

CONTIGUITY = [(∑^z^_r = 1_ *c_ijr)_/a^*^_ij_] − 1/v − 1, (A3)

c_ijr_ = contiguity value for pixel r in patch ij, v = sum of the values in a 3-by-3 cell template, a_ij_ = area of patch ij in terms of number of cells.

PROXIMITY=∑^n^_s = 1_(a_ijs_/h^2^_ijs_) (A4)

a_ijs_ = area (m^2^) of patch ijs within specified neighborhood (m) of patch ij, h_ijs_ = distance (m) between the patch and the focal patch of all patches of the corresponding patch type whose edges were within a specified distance (m) of the focal patch, based on patch edge-to-edge distance, computed from cell center to cell center.

SIMI=∑^n^_s = 1_(a_ijs_*d_ik_)/h^2^_ijs_, (A5)

a_ijs_ = area (m^2^) of patch ijs within specified neighborhood (m) of patch ij, d_ik_ = similarity between patch of types i and k, h_ijs_ = distance (m) between the patch and the focal patch of all patches of the corresponding patch type whose edges were within a specified distance (m) of the focal patch, based on patch edge-to-edge distance, computed from cell center to cell center.

**Table A1:** Spectral bands and indices of Landsat 8.

Item #	Variables	Description
June and September		
1	Band 1	Coastal aerosol
2	Band 2	Visible blue
3	Band 3	Visible green
4	Band 4	Visible red
5	Band 5	Near-infrared
6	Band 6	Short wave length infrared 1
7	Band 7	Short wave length infrared 2
8	NDVI	Normalized difference vegetation index
9	EVI	Enhanced vegetation index
10	NDMI	Normalized difference moisture index
11	SAVI	Soil adjusted vegetation index
12	MSAVI	Modified soil adjusted vegetation index
13	NBR	Normalized burn ratio
14	NBR2	Normalized burn ratio 2

**Table A2:** Edge depth matrix between groups of habitats used for calculation of core area index (meters).

Group #	1. Broadleaf: Highly Suitable	2. Birch with Larch: Moderately Suitable	3. Spruce with Birch and Fir: Low Suitability	4. Unsuitable: Farmland, Wetland, Settlement
1. Broadleaf: highly suitable	0	0	100	1000
2. Birch with larch: moderately suitable	0	0	100	1000
3. Spruce with birch and fir: low suitability	0	0	0	1000
4. Unsuitable: farmland, wetland, and settlement	0	0	0	0

**Table A3:** Contrast matrix between groups of habitats used for calculation of edge contrast index.

Group #	1. Broadleaf: Highly Suitable	2. Birch with Larch: Moderately Suitable	3. Spruce with Birch and Fir: Low Suitability	4. Unsuitable: Farmland, Wetland, Settlement
1. Broadleaf: highly suitable	0	0	0.5	1
2. Birch with larch: moderately suitable	1	0	0.2	1
3. Spruce with birch and fir: low suitability	0.5	0.2	0	0.2
4. Unsuitable: farmland, wetland, settlement	1	1	0.2	0

**Table A4:** Neighborhood matrix between groups of habitats used for calculation of similarity index.

Group #	1. Broadleaf: Highly Suitable	2. Birch with Larch: Moderately Suitable	3. Spruce with Birch and Fir: Low Suitability	4. Unsuitable: Farmland, Wetland, Settlement
1. Broadleaf: highly suitable	1	0.8	0.2	0
2. Birch with larch: moderately suitable	1	1	0.2	0
3. Spruce with birch and fir: low suitability	0.8	0.8	1	0.2
4. Unsuitable: farmland, wetland, settlement	0	0	0	1

**Table A5:** Fragmentation metrics of the selected 547 patches.

Fragmentation Metric (Mean Patch Value)	Class Metric Means *		
	Class 1	Class 2	Class 3
Number of patches	3	303	241
Area (hectares)	162,184.4	656.2	1375.8
Perimeter (km)	8694.0	52.0	102.9
Shape (normalized)	53.7	3.2	5.0
Contiguity(normalized)	0.87	0.37	0.57
Core area (hectares)	94,203.2	331.5	632.4
Proximity(normalized)	28,843.2	804.9	28,751.0
ENN (meters)	180.0	196.8	184.5
Edge contrast(normalized)	13.9	10.1	17.3
Similarity(normalized)	176,166	2,148,419	60,217
Total class area (hectares * 10^3^)	486.5	198.8	331.6

* 547 patches were classified into 3 classes by *k*-means method to demonstrate typical patterns and their spatially uneven distribution. Class #1 included three patches that occupied 486.5 thousand hectares in the southern part of Lesser Khingan. This class had the highest (best) metrics of fragmentation, except similarity and edge contrast. Class #1 can be considered as habitats with the largest capacity for wild ungulates, even though surrounded by settlements and farmland. Class #2 has a total area of 198.8 thousand hectares, and it has highest values of edge contrast and similarity. The patches of Class #2 are located in the north and central part of the Lesser Khingan. It consists of broadleaf forests, mainly situated in lowlands and midlands. It has a moderate capacity for ungulates, due to the small area of broadleaf forests but, at the same time, it has good connectivity between patches. Human activity is minimal for the Class #2. Class #3 has an area of 331.6 thousand hectares. Compared to Class #2, it is characterized with 1.5–2 times higher metrics meanings in core area, perimeter, and shape, but it has the lowest values of edge contrast and similarity. This can be explained by the location of patches in either anthropogenic fragmented areas of central Lesser Khingan or in separated forest territories in the Lesser Khingan surroundings.
